# Knowledge, attitudes, and behaviours towards smoking among people with migration experience: a global scoping review

**DOI:** 10.1186/s12889-025-24258-y

**Published:** 2025-09-30

**Authors:** Kris Schürch, Sophie Meyer, Marina Köhli, Beatrice Minder, Doris Kopp-Heim, Magda Gamba, Christoph Buhne, Daniel Ludin, Jodie Freeman, Blender Muzvondiwa, Lisa M. Held, Octavio Pano, Cristopher I. Kobler Betancourt, Lucia Bühlmayer, Harvy Joy Liwanag, Annika Frahsa

**Affiliations:** 1 Institute of Social and Preventive Medicine, University of Bern, Bern, Switzerland; 2 Graduate School for Health Sciences, University of Bern, Bern, Switzerland; 3 Swiss Association for Tobacco Control (AT Switzerland), Bern, Switzerland; 4 Public Health & Primary Care Library, University Library of Bern, University of Bern, Bern, Switzerland; 5 Central and University Library of Lucerne, Lucerne, Switzerland; 6 Faculty of Health Sciences and Medicine, University of Lucerne, Lucerne, Switzerland; 7 School of Public Health, University at Albany, Albany, NY USA; 8 Translational Neurotechnology Laboratory, Department of Neurosurgery, Klinikum Rechts Der Isar, Technical University of Munich, Munich, Germany; 9 Division of Preventive Medicine, Brigham and Women’s Hospital, Boston, MA USA; 10 Harvard Medical School, Boston, MA USA; 11 Saw Swee Hock School of Public Health, National University of Singapore, Singapore, Singapore

**Keywords:** Migrants, Global smoking among migrants, International comparisons, KAB

## Abstract

**Background:**

Knowledge, attitudes, and behaviours towards smoking have been extensively researched across diverse populations with migration experience. The objective of this scoping review was to understand the extent, type, and geographical distribution of the published literature on smoking among people with migration experience across the globe.

**Methods:**

We conducted a scoping review using the Joanna Briggs Institute methodology. Supported by two information specialists, we performed a comprehensive literature search (from 2012- 17 January 2024) in five databases, without language or geographic restrictions. The search yielded 8,400 potentially relevant records after deduplication. After title and abstract screening, 305 full texts were included for descriptive analysis and out of these, 25 that covered all three aspects (knowledge, attitudes, and behaviour) were included for content analysis.

**Results:**

The review identified a predominance of quantitative studies (87%), with a smaller proportion of qualitative (12%) and mixed-methods studies (2%). Most studies focused on behavioural prevalence related to tobacco and nicotine product consumption, with 72% addressing behaviours specifically, though only a subset (26%) focused directly on tobacco and/or nicotine use among migrants. Geographical analysis revealed that most of the research originated from high-income countries in particular the USA (*n* = 126), Canada (*n* = 32), and Germany (*n* = 20), with notable gaps in regions with significant migrant populations, such as Saudi Arabia (*n* = 1). Among the 25 KAB-focused studies, data collection was based on surveys (52%), interviews (40%), and focus groups (28%). Findings showed varying knowledge of tobacco harms, shaped by socio-economic status, acculturation, and health literacy. Attitudes were influenced by culture and religion, while smoking behaviours were driven by gender, stress, peer influence, and migration-related pressures. Terminology varied across studies, with inconsistent definitions for key terms "migrants" and "immigrants," complicating comparisons between populations and countries. Definitions of tobacco products also varied across studies.

**Conclusions:**

This scoping review reveals significant gaps in research on migrants' knowledge, attitudes, and behaviours towards tobacco and nicotine consumption, including a lack of qualitative studies, inconsistent terminology, and a geographic focus on high-income countries. Addressing these gaps through expanded research in underrepresented regions and standardising terminology is essential for developing culturally relevant public health strategies.

**Supplementary Information:**

The online version contains supplementary material available at 10.1186/s12889-025-24258-y.

## Background

As of 2020, the United Nations estimated that there were 281 million international migrants, comprising 3.5% of the global population [1]. People migrate for many reasons, whether for better lives or new opportunities [2]. One notable trend is the increasing number of migrants being displaced from non-European countries, either within or outside of their countries, due to conflict or economic instability. Many of these migrants move to countries in the Americas and Europe. The European Union (EU) as such has been described as a “global migration magnet”, drawing unprecedented numbers of migrants, refugees, and asylum seekers [3, 4]. During what has been termed the “migration crisis” of the last decade, 1.9 million people migrated into the EU from non-member countries in 2014, while 1.8 million moved between member states [3]. By 2022, the International Organization for Migration (IOM) reported 117 million displaced people worldwide, including 71.2 million internally displaced individuals, with the number of asylum-seekers increasing by over 30% [5]. These demographic shifts have resulted in highly heterogeneous migrant populations across the globe with diverse cultural identities and health behaviours. Migration itself has become a determinant of health, affecting access to the healthcare system and directly impacting the physical and mental health, and well-being of migrants, as highlighted by the COVID-19 pandemic [6–8]. Additionally, migration influences changes in health behaviour, in particular smoking [9].

Smoking stands out as a critical public health issue due to its status as one of the leading causes of premature mortality and reduced quality of life [10]. Smoking is known to cause a variety of cancers and cardiovascular diseases, with prevalence varying widely based on socioeconomic status, education, stress, age, and gender [11–14]. In the context of migration, people’s smoking behaviours are also influenced by the prevailing norms and attitudes both in the country of origin and the host country. For instance, as migrants move to regions with tobacco control measures different from those in their country of origin, their smoking patterns may shift significantly [15–25].

Currently, there is a body of research on the Knowledge, Attitudes and Behaviours (KAB) of various migrant populations towards smoking (see Table 1 for definitions) [32–38]. KAB research in the context of smoking is key for developing effective public health interventions and policies. Understanding the knowledge component may help identify gaps in awareness about the risks associated with smoking [39]. Analysing attitudes provides insights into the underlying beliefs and perceptions that may influence smoking behaviour, including the social and cultural norms accompanying them [40]. Examining behaviours reveals smoking patterns that may be linked to knowledge, attitudes and vice-versa. For example, social groups and peers play an important role as they link behaviour with identity and attitudes, in some cases promoting use, while in others deterring it [41]. Together, KAB studies offer comprehensive frameworks for designing targeted public health strategies.

**Table 1:** Definition of terms

Migrant	A person who is moving or has moved across an international border or within a state away from their habitual place of residence, regardless of the person’s legal status, whether the movement is voluntary or involuntary, what the causes for the movement are and what the length of the stay is [26].
Tobacco and/or nicotine products	The consumption of tobacco and nicotine products most often involves the act of inhaling and exhaling the aerosols of a substance. This act is commonly associated with burning tobacco, smoked in the form of a cigarette, cigar or pipe, but is not limited to these materials and can also include the heating of e-liquids within e-cigarettes, or other ENDS (electronic nicotine delivery systems). Under this concept, we also include smokeless products, such as snus, or nicotine pouches [27].
Knowledge (K)	Embodies “all information that a person possesses or accrues related to a particular field of study” [28]. For smoking, this can be in regard to knowledge of harmful effects, or perception of health effects of smoking (e.g. “smoking is harmful”, “smoking can cause heart diseases”) [29].
Attitudes (A)	Refers to the subjective sum all feelings and outlooks toward a particular concept, idea or action [30]. Attitudes can be a belief or idea associated with a particular object, can represent the individual’s evaluation and emotion associated with the object, or attitudes can represent the predisposition of action towards the object [28]. Smoking related attitudes include “smoking is pleasurable”, “smoking relaxes me”, “smoking helps me lose weight” [31].
Behaviours (B)	Are understood as an observable action. In other words, the way a person, or group of persons act in certain conditions [28]. Smoking related behaviours include “tobacco use among adults”, “daily hookah smoking youth”

Several reviews on migrant health have also been published, each focusing on different aspects of health or different migrant groups, such as migrant workers [42–45]. The studies included in these reviews show variability in the types of migrants researched and how they are defined. In one study, the terms refugees and migrants were grouped as “non-permanent residents”, yet the differences between refugees and migrants remain unclear [46].

While there have been some related reviews on smoking among migrant populations, to our knowledge, this is the first review to specifically explore the KAB towards smoking amongst migrants with such a global scope [24, 47].

Therefore, the main objective of this study was to present an updated landscape of research on smoking among various migrant populations across the globe. Through this analysis, we aim to provide a nuanced understanding of the breadth and depth of existing evidence on this critical issue, while highlighting challenges and opportunities for future research amongst migrant populations and their KAB towards smoking.

Specifically, we sought to analyse the literature in relation to: (a) study designs and methodologies; (b) KAB and focus on consumption of tobacco and nicotine products; (c) Geographical differences in host countries studied; and (d) Terminology used to refer to migrants and smoking.

## Methods

We developed a scoping review protocol in accordance with the Joanna Briggs Institute (JBI) methodology for scoping reviews [48] that is publicly available elsewhere. The protocol includes the initial MEDLINE search. This scoping review followed the Preferred Reporting Items for Systematic reviews and Meta-Analyses extension for Scoping Reviews (PRISMA-ScR) guidelines (see Additional file 1).

In response to the challenges of terminology, and for the purpose of this study, we employed a broad definition of “migrant” to encompass various terms for the scoping review, referring to the IOM definition (see Table 1) [49]. This approach was similarly taken by Villarroel et al*.*(2019) for their scoping review on migrant health research [43]. We also consulted the World Health Organization (WHO) website for a comprehensive description of smoking that encompasses products beyond traditionally smoked tobacco [26]. We therefore expanded smoking to include oral tobacco and the use of nicotine products, such as e-cigarettes. This approach reflects the diverse range of tobacco and nicotine products available today and ensures the inclusion of research focusing on non-traditional smoking products [27]. Finally, we based our KAB definitions on Schrader & Lawless (2004) (see Table 1), also acknowledging that KAB components may overlap and influence each other [28].

## Search strategy

A comprehensive literature search was conducted in five bibliographic databases [Medline, Embase.com, Web of Science Core Collection, PsycINFO (Ovid), and Global Health (Ovid)] until January 17, 2024, with the assistance of two medical information specialists. To identify relevant text words and controlled vocabulary (also called index or MeSH terms), we started an initial search on Medline (Ovid) based on the IOM definition of migrants and refined it using a test set of 10 core articles provided by the first author. Articles such as Castafieda (2022), and Galea, Ettman, & Zaman (2022) provided common juridical categories—immigrant, migrant, refugee, asylum seeker, or internally displaced person—with each label situated on a continuum marked by two major dimensions: voluntary (economic immigrant, migrant worker) and involuntary departure (refugee, asylum seeker, internally displaced person).” [3, 9, 26] We therefore considered these types of migrants on the continuum in the review.

We limited the search to the last 12 years to capture the latest tobacco and nicotine product developments and ensure that the review remains relevant to today’s research [50]. Animal studies, clinical trials, letters, editorials, or congress abstracts were excluded. The Medline search strategy was appropriately translated to Embase.com, Web of Science Core Collection, PsycINFO (Ovid), and Global Health (Ovid) (see Additional file 2 for full electronic search strategy used for each database). We applied no language and geographical restrictions. Duplicate records were removed using Deduklick (Risklick AG, Bern, Switzerland), a fully automated and validated deduplication software which was found to be comparable to manual deduplication [51].

### Screening process

All references from the initial searches were exported from the databases to Endnote20 (Clarivate Analytics, PA, USA), which was used by at least two reviewers to independently screen each reference by title and abstract. Discrepancies were resolved through discussion between the first author and the reviewers. Some of the title and abstract screening for newly identified references and full-text screening were conducted on the Covidence platform (www.covidence.org) (Covidence, Melbourne VIC, Australia) for an efficient and collaborative review. The appropriateness of the selected evidence was determined based on pre-defined inclusion criteria, which were established through an iterative process. The final inclusion criteria are shown in Additional file 3.

### Data extraction and data analysis

The data extraction tool was also developed as part of an iterative process, with discussions among the core group to identify key information to be extracted from the articles with reference to previous work by Arshad, Matharoo, Arshad, Sadhra, Norton-Wangford, & Jawad (2019) and Selamoglu, Erbas, Kasiviswanathan, & Barton (2022) [9, 52]. Additionally, Cattacin [53], Ranci [54], Walker & Fox [55], and Aslany, Carling, Mjelva, & Sommerfelt [2] informed the further development of the data extraction tool, including whether the study considered contextual factors, or the roles of social dynamics and other lifestyle factors when researching the KAB of tobacco and/or nicotine consumption amongst migrants. We pilot-tested the data extraction tool on the Covidence platform with 15 studies, during which we observed there were studies in our list where the consumption of tobacco and/or nicotine products was not the primary focus. We subsequently adjusted the extraction tool to allow the categorisation of the relevance of tobacco and/or nicotine products in each study. Specifically, we distinguished between studies where the consumption of these products was the primary focus and studies where smoking was only part of describing participants' health profile rather than as the primary topic of investigation. At least two reviewers performed data extraction on Covidence, while the first author resolved any discrepancies between the two sets of extracted information. The data extraction tool is in Additional file 4.

Descriptive analysis of the extracted information was performed through figures and tables created on Microsoft Excel 2024 (Microsoft, Redmond WA, USA) and Adobe Illustrator 2024. We performed a content analysis of the subset of studies that encompassed all three KAB aspects and which primarily focused on the consumption of tobacco and/or nicotine products (*n* = 25, see Table 3).

## Results

**Fig. 1:**
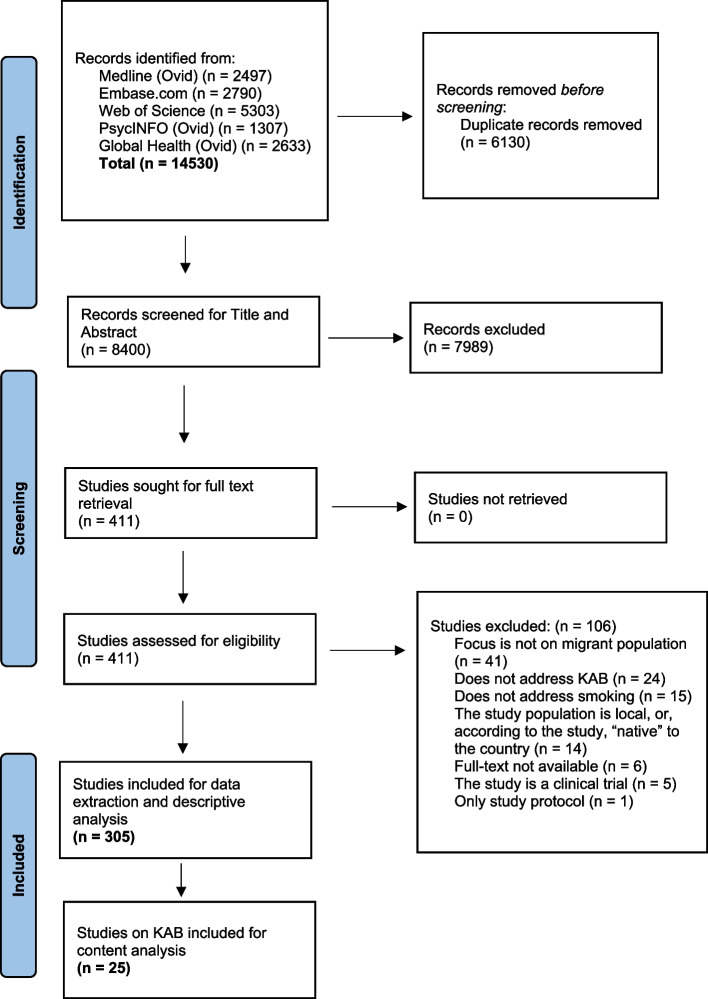
PRISMA 2020 flow diagram of steps taken for research on smoking amongst migrants

Figure 1 illustrates our PRISMA flowchart with regard to Page et al. [56]. The initial database search yielded 14′530 records. After removing duplicates, we identified 8′400 potentially relevant records. Following title and abstract screening, we included 305 publications on KAB related to the consumption of tobacco and/or nicotine products amongst migrants (see Additional file 5 for the full list of included studies and extracted data).

### Timeline of publications

The trend in the number of publications over time showed fluctuations from 2012 to 2023, with peaks in 2012 (*n* = 33), 2015 (*n* = 31), and 2018 (*n* = 33). and a rise in 2022 (*n* = 28).

### Study designs and methodologies

There was a predominance of quantitative research methods, accounting for *n* = 265 (87%) of included studies (see Table 2). Qualitative studies comprised *n* = 35 (12%), while mixed methods studies represented *n* = 5 (2%) of the included studies.

**Table 2:** KAB and focus on consumption of tobacco and/or nicotine products

Study KAB focus	Count (% of total)	With focus on consumption of tobacco and/or nicotine products (% of total)	Without focus on consumption of tobacco and/or nicotine products (% of total)
Knowledge and attitudes	3 (1%)	2 (0.7%)	1 (0.3%)
Only attitudes	3 (1%)	2 (0.7%)	1 (0.3%)
Only knowledge	3 (1%)	1 (0.3%)	2 (0.7%)
Knowledge and behaviours	13 (4%)	6 (2%)	7 (2%)
**Knowledge, attitudes, and behaviours**	**25 (8%)**	**25 (8%)**	**0 (0%)**
Attitudes and behaviours	37 (12%)	26 (9%)	11 (3.6%)
**Only behaviours**	**221 (72%)**	**80 (26%)**	**141 (46%)**
**Total**	**305 (100%)**	**142 (47%)**	**163 (53%)**

Table 2 shows the distribution of studies on Knowledge, Attitudes, and Behaviours (KAB) related to tobacco and/or nicotine consumption among migrants. Categories in bold—studies focusing solely on behaviours (B) and those covering all three KAB aspects—highlight the predominance of behaviour-focused studies. The 25 studies addressing all three KAB components were selected for content analysis to offer deeper insights into this subset

### KAB and focus on the consumption of tobacco and/or nicotine products

Among the 305 included studies, we found only *n* = 25 (8%) studies that assessed all three KAB and also had a focus on the consumption of tobacco and/or nicotine products. Furthermore, n = 221 (72%) of included studies represented behaviour-specific prevalence studies, of which *n* = 141 (46%) did not primarily focus on the consumption of tobacco and/or nicotine products but instead presented results from broader health surveys, such as health monitoring or lifestyle factor assessments. The assessment of current smoking behaviour status also varied across studies. Some studies based the assessment on self-identification as “current smoker” without further differentiation, typically recorded as a yes/no response to “Do you currently smoke?”. Other studies defined “current smoker” based on either the person “smoked in the last 30 days” or “having smoked at least 100 cigarettes in their lifetime, and now smoking every day or some days of the week”.

The studies varied in their approaches to assess KAB related to the consumption of tobacco and/or nicotine products depending on the contexts, the target migrant populations, and studies’ specific research aims. For instance, the study by Tirukkovalluri [57] applied the Global Adult Tobacco Survey version 2.1 to assess socio-demographic profile, tobacco use status in any form, as well as knowledge, attitudes and perceptions to tobacco cessation and tobacco harms among interstate migrant construction workers. Jiménez-Muro [58], on the other hand, assessed the knowledge of whether smoking during pregnancy affects the foetus, as well as the attitudes towards the perception of risk amongst immigrant women (see Additional file 6).

### Geographical differences in host countries studied

**Fig. 2:**
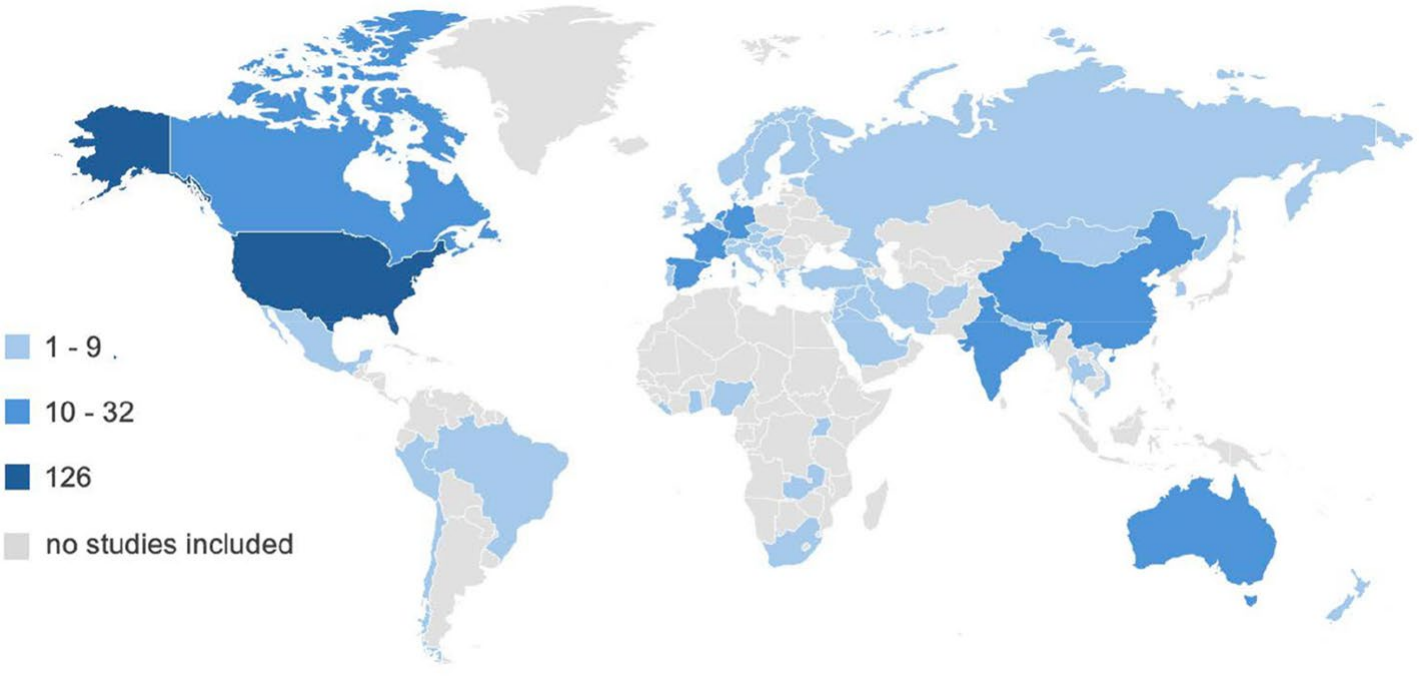
Geographical mapping of the countries hosting migrants in the included studies. Note: Fig. 2 illustrates the geographical distribution of studies on tobacco and/or nicotine consumption among migrants, highlighting the host countries where research was conducted. The numbers include cumulative mentions of individual studies from literature reviews

Included studies represented 60 different countries hosting migrant populations, with the USA as the host country from which most studies (*n* = 126, 32%) had been published, followed by Canada (*n* = 32, 8%), Germany (*n* = 20, 5%), and the Netherlands (*n* = 18, 5%) (see Fig. 2 and Table 3). 22 studies focused on more than one country by reviewing the literature or comparing smoking prevalences between two countries up to 24 countries [59]. European or North American countries were present in all review and comparison studies (*n* = 22, 100%), while only a fraction of these studies included an Asian (*n* = 6/22, 27%), Middle Eastern (*n* = 6/22, 29%), African (*n* = 5/22, 23%), or South American country (n = 3/22, 14%). Of all included studies, 132 (43%) had a national level of inquiry, which is also associated with the most used study method: quantitative studies that often used data from national surveys. 65 (21%) studies specified a city, 58 (19%) specified a region, 28 (9%) a specific community or neighbourhood, and 22 multi-country international studies.

**Table 3:** Top 10 most frequent migrant host countries studied

Country	Number of studies
1) USA	126
2) Canada	32
3) Germany	20
4) Netherlands	18
5) China	16
6) Australia	15
7) India	11
7) France	11
9) Spain	10
10) UK	9
10) Israel	9
10) Sweden	9

Table 3 lists the top ten host countries most frequently studied in research on tobacco and/or nicotine consumption among migrants. The numbers include cumulative mentions of individual studies from literature reviews

### Terminology used

#### Focus on migrant group

Most studies included had a specific focus on researching [individual] migrants, or groups of migrants (*n* = 176, 58%). In the other 42% (*n* = 129) of studies, analyses compared migrants, or groups of migrants, with Indigenous populations of the respective country.

#### Main migrant groups studied

**Fig. 3:**
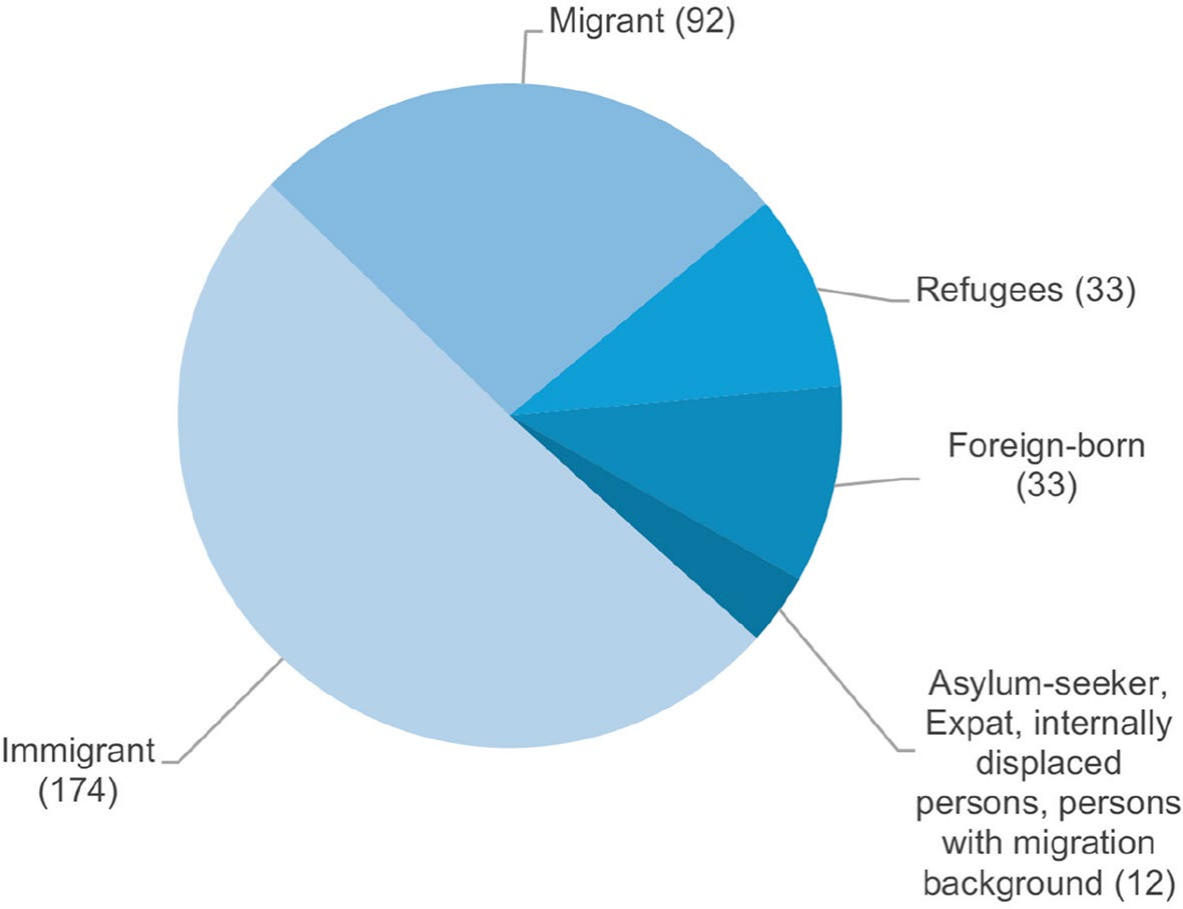
Terminology used to describe migration process (non-mutually exclusive). Note: Fig. 3 illustrates the terminology used to describe migration processes in the included studies, showing the frequency of different terms used. The figure highlights significant variability in terminology, reflecting inconsistencies in how migrant populations are defined across studies

The most frequently used term was 'immigrants' (*n* = 174, 50%). Many studies did not consistently use a single term throughout. To clarify, we counted the presence of each distinct term used to describe migrant groups in the 305 included papers, resulting in more than 305 mentions (see Fig. 3). If a paper used multiple terms, such as both 'immigrant' and 'migrant' to describe the same group, each term was counted once per paper. Moreover, the terms’ use and definitions varied between papers.

### Content analysis of KAB studies

#### Measurements of KAB in the studies

Data on KAB were collected from multiple sources across the 25 KAB studies (see Additional file 6 for a detailed overview and spotlight on 25 KAB studies). Questionnaires or surveys were used in *n* = 13 studies (52%). Interviews (*n* = 10, 40%) were frequently paired alongside focus groups (*n* = 7, 28%). One mixed methods study (Tregobov et al., 2020) collected data with interviews, focus groups and surveys [60].

#### Patterns and context-specific differences in KAB

Several patterns and context-specific differences emerged in knowledge, attitudes, and behaviours related to tobacco use among migrant populations (see Table 4).

**Table 4:** Spotlight on 25 KAB studies

Author(s), year	Term used	Definition/description of migrant population	Outcomes reported	Products studied	Main findings	Is the study considering factors apart from migration, or migration background?
Castellanos 2011 [61]	Latinx or Hispanic Immigrants	Self-identified as Latino or Hispanic, or born in a Latin-American country or who have a parent or grandparent born in a Latin-American country	K:	Cigarettes	Latino smokers in Minnesota showed stage-specific differences in beliefs, behaviors, and readiness to quit. Smokers in the maintenance stage were older, more likely to be married, and had more restrictive smoke-free environments. Those in preparation stages were less likely to enjoy smoking, more optimistic about quitting, and less exposed to secondhand smoke than those in pre-contemplation. Never smokers reported better health, less exposure to stress, and stronger anti-smoking attitudes. Social and environmental factors—like exposure to secondhand smoke, belief in willpower, and support networks—significantly influenced smoking status and cessation intentions	Country or community of origin; Subjective individual factors (social identity, behavioural factors); Lifestyle factors (alcohol); Acculturation; Years in Host country, Household composition, age of entry in US
			Knowledge of harms of tobacco			
			A:			
			Attitudes to smoking cessation and addiction, smoking for stress			
			B:			
			Frequency of smoking, addiction, use of other tobacco, household rules around smoking			
Zou 2019 [62]	Migrants	Not explicitly defined. Described as"Nearly 70% had left their hometown for more than 10 years."	K:	Cigarettes	Implementation of smoking cessation pilot was influenced by societal norms (e.g., smoking as a social custom), individual-level barriers (e.g., low education, mobility, weak health beliefs), and programmatic challenges (e.g., limited influence of trained team leaders due to workload and weak authority). While team-led counseling and WeChat-based support were positively received, sustained cessation was hindered by social pressure, limited comprehension of health messages, and relapse risks, especially among less motivated or isolated workers	Socioeconomic status (poverty, education, employment); Sex/gender (male vs female); Acculturation
			Smoking-related health knowledge			
			A:			
			Influence of social traditions on smoking, attitudes towards smoking and smoking cessation			
			B:			
			Smoking habits			
Wilhelm 2022 [63]	Somali refugees	Reporting foreign birth and identifying as ethnically Somali	K:	Waterpipe/shisha; Other tobacco use not specifically defined	Informants reported increased tobacco use—especially shisha—among Somali adolescents in the U.S., driven by evolving social norms, low risk perceptions (especially about shisha), and ambiguous religious messaging. Families were seen as key to prevention but lacked awareness and tools, particularly regarding newer tobacco products. Faith leaders and culturally congruent mentors were identified as valuable supports, while clinics faced barriers in engaging Somali youth. Youth engagement in prevention strategy design was also emphasised	Country or community of origin; Social dynamics (stigma, cultural practice/rituals)
			Risks associated with tobacco use, especially shisha			
			A:			
			Somali adolescent tobacco prevention compared with other issues, perception of risks, especially shisha, role of norms and increasing exposure to tobacco products in the United States			
			B:			
			Factors influencing Somali youth tobacco decision making, and primary community assets and barriers related to adolescent tobacco use and prevention efforts with a particular focus on the parent–child relationship, faith influences on tobacco use			
Wilhelm 2022 [64]	Somali refugee diaspora	Reporting foreign birth and identifying as ethnically Somali	K:	Waterpipe/shisha; Other tobacco use not specifically defined	Key informants reported that Somali immigrant parents in the U.S. face challenges in addressing adolescent tobacco use due to structural barriers (e.g., language, unfamiliarity with institutions), cultural clashes, shifting parental roles, and acculturative gaps. These factors weaken traditional parental authority and monitoring. Parents often lack knowledge about tobacco products and feel unprepared to prevent youth use. Informants emphasised the need for culturally appropriate support to help Somali parents adapt their parenting and communication strategies for effective tobacco prevention	Social dynamics (stigma, cultural practice/rituals); Acculturation, Parenting experiences in Somalia vs US
			Knowledge of U.S. tobacco products and modalities, and about the environments in which their children might be exposed to tobacco product			
			A:			
			Somali parents’ preparedness to address adolescent risk behaviours such as tobacco use			
			B:			
			Somali parents'influence on children's tobacco use, role of acculturation on structural challenges and multicultural identity formation			
Urban 2015 [65]	Bosnian, Turkish migrant smokers	Turkish or Bosnian migration background—following the definition of the “Recommendations for the 2010 censuses of population and housing” of the United Nations Economic Commission for Europe (UNECE), requiring that both parents were born abroad	K:	Cigarettes; Other tobacco/smoking consumption not specifically defined	Bosnian smokers had the highest nicotine dependence (Fagerström score 4.7), followed by Turkish (4.0) and Austrian (3.4). Migrant smokers were more willing to quit and had more cessation attempts, but received less cessation support, especially Turkish migrants. Migrants preferred group counselling, native-language services, and religious venues for cessation, unlike Austrians who favored individual counselling and medical settings	Country or community of origin
			Awareness of tobacco-related diseases			
			A:			
			Attitudes towards smoking cessation (preferred method, who should assist, preference for single or group counselling, preferred cessation language)			
			B:			
			Age of first cigarette, daily cigarettes, frequency of smoking. Various characteristics of smoking cessation (attempts, how often, which method)			
Tregobov 2020 [60]	Chinese-Canadian immigrants	Chinese descent (Mandarin- or Cantonese-speaking), who had immigrated to Canada within the past 5 years or were the children of Chinese immigrants to Canada	K:	Cigarettes	Smoking initiation was influenced by peer pressure, family role modeling, and stress, particularly from immigration. Cultural norms promoted smoking as a social tool, especially among men, while women faced stigma that discouraged both smoking and seeking cessation support. Cessation barriers included limited access to linguistically appropriate resources, gender-based stigma, and low perception of health risks. Motivators to quit included family concern, smoke-free laws, and desire to protect children from secondhand smoke	Social dynamics (stigma, cultural practice/rituals); Sex/gender (male vs female), Stress, family influence, work environment, government regulations
			Knowledge of factors influencing smoking behaviours			
			A:			
			Views on a number of environmental, cultural, and personal factors influencing smoking onset, continuation, and cessation			
			B:			
			Smoking patterns, facilitators or barriers to quitting smoking			
Tirukkovalluri 2020 [57]	Interstate migrant construction workers in Chennai	Not explicitly defined	Use of Global Adult Tobacco Survey Version 2.1	Tobacco use not specifically defined	98% currently used tobacco—primarily smokeless forms (58.6%). Despite 84.6% awareness of health harms, only 27.5% had visited a doctor in the past year, and just 25.1% had attempted quitting. Pictorial health warnings were widely noticed (91.3%) but influenced quitting intention in only 31.9%. Only 1.17% had used cessation aids. Migrants using tobacco for < 5 years were more likely to try lower-risk alternatives (*p* = 0.001), while married and daily users were less likely to intend quitting	Socioeconomic status (poverty, education, employment) Marital status, years of consumption, duration of migration
			K:			
			Knowledge about tobacco harms. Awareness of lower risk alternatives			
			A:			
			Attitudes and perceptions about tobacco harms. Intention to quit in the future			
			B:			
			Current and former tobacco use, form of tobacco and frequency of use. Cessation attempts			
Onigbogi 2015 [66]	Migrants from northern Nigeria	Residence within the Idi-Araba community over the past 3 months prior to the study, an area where there is a high concentration of migrants	K:	Menthol cigarettes	Among 24 male migrant workers in Lagos, smoking was driven by peer pressure, social belonging, perceived adult status, and stress relief. 62% smoked first cigarette within 30 min of waking up. Smoking was seen as conferring higher status, and increasing alertness. This was especially relevant with mentholated cigarettes. Barriers to quitting included lack of institutional support, reliance on self-management (e.g., prayer, fasting), and social reinforcement of smoking behaviors	Socioeconomic status (poverty, education, employment), Ethnicity, marital status, age
			Effects of smoking mentholated cigarette brands			
			A:			
			Reasons for initiating and continuing to smoke cigarettes. Attitudes towards barriers to quitting			
			B:			
			Smoking initiation and smoking behaviour and factors affecting brand choices. Behavioural barriers to quitting			
Nguyen 2018 [67]	Foreign-born	Respondents were categorized as foreign-born if they answered no to the question"Were you born in the United States?”	K:	Cigarettes; Nicotine	Foreign-born individuals were significantly less likely to be smokers and more likely than U.S.-born respondents to be concerned about nicotine addiction and to believe that low nicotine cigarettes (LNCs) had significantly lower cancer risk. Among foreign-born groups, non-Hispanic Black and Hispanic respondents were more likely than non-Hispanic Whites to believe that LNCs were more harmful and more addictive. Lower acculturation (measured by English proficiency and time in the U.S.) was associated with greater misperceptions about nicotine’s role in cancer and addiction	Country or community of origin; Socioeconomic status (poverty, education, employment); Sex/gender (male vs female); Acculturation, Race/ethnic identity, age, marital status
			Knowledge about nicotine, addiction, and causes of smoking-relating cancers			
			A:			
			Beliefs about nicotine and concerns about nicotine addiction. Perception of cigarette addiction. Beliefs about low nicotine cigarettes			
			B:			
			Current smoking status			
Mukherjea 2012 [68]	South Asians immigrants (India, Bangladesh, Pakistan, Sri Lanka)	Ethnic identification as South Asian	K:	Waterpipe/shisha; Oral tobacco (e.g. snus); Bidi	Among 88 South Asian participants in the U.S., culturally-specific tobacco products like bidis, paan, gutkha, and hookah were widely used. Knowledge of health risks was often incomplete or inaccurate. Many perceived these products to have health benefits (e.g., aiding digestion, providing calcium). Tobacco use was linked to preserving tradition, cultural celebration, and expressing ethnic identity. Use patterns reflected social hierarchy, with more discreet use by women and younger individuals. Products were readily accessible in ethnic outlets, often without proper labeling or regulation	Country or community of origin; Subjective individual factors (social identity, behavioural factors); Sex/gender (male vs female), duration of residence in the US
			Health consequences of tobacco use			
			A:			
			Reasons for and connotations attached to different use of specific products mentioned, attitudes towards social and cultural value of tobacco use (e.g. traditional customary aspects of ethnicity)			
			B:			
			Spectrum of used products, consumption settings (e.g. hidden)			
Mehra 2020 [69]	Male Afghan migrants	Not explicitly defined. Participants described as stemming from local Afghan community	K:	Cigarettes; Waterpipe/shisha	70% smoked tobacco (55.9% used cigarettes and 13.6% used Naswar). 89% used non-Indian tobacco products, often procured via friends and family. High cost (85%), and limited availability (19%), as well as stricter Indian tobacco laws (50%) were seen as barriers to use. Only 23% admitted to be unaware of the ill effects cause by tobacco, yet 75% did not want to quit, likely due to 58% fearing to Loose friends, and 92% were unaware of how to access cessation services	Country or community of origin; Social dynamics (stigma, cultural practice/rituals); Sex/gender (male vs female), Current tobacco control policies in place
			Knowledge of harmful effects of tobacco, knowledge of cessation services			
			A:			
			Attitudes towards barriers for tobacco use in India, and barriers to quitting tobacco			
			B:			
			Current and previous tobacco usage patterns and practices, types of tobacco products used, procurement of tobacco products			
Lo 2016 [59]	Internally/forcibly displaced peoples; Refugees	Not explicitly defined	K:	Cigarettes; Waterpipe/shisha; smokeless tobacco	From 39 included studies, tobacco use prevalence among conflict-affected civilians varied widely (e.g., 4.7% to 70.7%). Tobacco use was commonly higher among males, adolescents, and individuals with post-traumatic stress disorder (PTSD) or depression. Tobacco served as a coping mechanism for trauma, stress, and displacement. Evidence on how conflict directly affects tobacco use was mixed. Only one study measured cessation rates, showing low success. No studies assessed the effectiveness of tobacco interventions in these populations. Quality of most studies was moderate or weak	exposure to armed conflict
			Awareness of negative health impacts of smoking. Knowledge of cessation and barriers to cessation			
			A:			
			Attitudes and perceptions towards smoking (smoking makes you look cooler, have more friends etc.). Reasons for smoking and motivations and barriers for cessation			
			B:			
			Smoking cessation rates and current tobacco use			
Liu 2015 [70]	Rural-to-urban migrant workers	Defined as an individual who was registered at a rural residence, had been working in the Urban context for at least 6 months without obtaining permanent residence	K:	Smoking not specifically defined	45.0% of males and 2.0% of females were current smokers. Risk factors for women included working in construction, entertainment, or restaurants, higher income, psychological distress (SCL-90 > 160), and multiple migratory cities. Among men, significant predictors included construction or entertainment work, being divorced/widowed, Longer migration duration, multiple migratory cities, and poor mental health. Over half of smokers reported workplace smoking; 58.8% had tried quitting. Smoking knowledge was Lower among smokers; 68.6% recognized its harms vs. 86.4% of non-smokers	Socioeconomic status (poverty, education, employment); Sex/gender (male vs female), Age, Marital status, Duration of migration (years), number of migratory cities
			Smoking and passive smoking are harmful to health			
			A:			
			Attitudes toward smoking and public smoking bans: negative, positive, neutral. Intention to quit			
			B:			
			Smoking-related behaviours: current smoking status, second-hand smoke exposure, prevalence, cumulative smoking time, daily cigarettes, monthly smoking expenditure, smoking locations. Odds ratio of current smoking by gender			
Lei 2021 [71]	Chinese immigrants	Not explicitly defined. Inclusion criteria were studies on chinese immigrants in the US	K:	Smoking not specifically defined	This review of 11 studies found that Chinese immigrants’ smoking behaviors were shaped by personal traits (age, education, gender), psychological stress, acculturation, and external cues (e.g. social norms). Barriers to cessation included language difficulties, low use of cessation aids, insufficient healthcare support, lack of social support, and misbeliefs (e.g., quitting is purely willpower-based). Lung cancer screening awareness was low, hindered by cultural and structural barriers	Socioeconomic status (poverty, education, employment); Social dynamics (stigma, cultural practice/rituals); Sex/gender (male vs female); Acculturation, Psychological status, language
			Knowledge and misconceptions of lung cancer screening and smoking cessation programmes			
			A:			
			Perceived blame and stigma associated with lung cancer and smoking, and preventive measures and how these may be influenced by language barriers and cultural beliefs such as'yin and yang'and'qi'. Personal beliefs towards smoking cessation			
			B:			
			Factors influencing smoking behaviours among Chinese immigrants. Reluctance to use cessation aids			
Lei 2021 [71]	Chinese immigrants	Defined as a person having origins in China	K:	Cigarettes	Among 10 Chinese immigrant smokers (7 men, 3 women), smoking behavior change followed three phases: beginning to smoke (often during adolescence via peer influence), maintaining smoking (linked to social norms, stress, and concealment), and changing smoking behavior (triggered by life events, U.S. smoke-free norms, or concern for loved ones). Participants cited boredom as a major cause of relapse and relied mostly on personal willpower rather than cessation aids	Socioeconomic status (poverty, education, employment); Sex/gender (male vs female); Acculturation
			Knowledge and impact of smoking behaviour change. Identification of barriers to quitting			
			A:			
			Personal opinions on smoking, and experiences with quitting, including the use of cessation methods			
			B:			
			Smoking history, experiences, and factors influencing their smoking habits before and after immigrating to the United States			
Kim 2012 [72]	Vietnamese American immigrants	Self-identification as Vietnamese descendants	K:	Cigarettes	24.4% of men and 1.2% of women were current smokers. Of 20 current smokers, 85% had used cessation medications, and 80% had attempted quitting in the past year. Acculturation was the only significant predictor of smoking—less acculturated men were over 5 times more likely to be ever-smokers. Knowledge of smoking harms was high across the sample, but attitudes toward smoking varied by education and age, not by smoking status	Socioeconomic status (poverty, education, employment); Subjective individual factors (social identity, behavioural factors); Sex/gender (male vs female); Acculturation
			Knowledge of the adverse health effects of smoking			
			A:			
			Attitudes toward smoking and seeking help for quitting			
			B:			
			Smoking and quitting behaviours			
Jiménez-Muro 2012 [58]	Immigrants	Immigrants were considered to be persons born outside Spain and their origin was grouped into Asia, Latin America, sub-Saharan Africa, Europeand Maghreb	K:	Tobacco consumption not specifically defined	Among 2,440 pregnant women in Zaragoza, 31.1% smoked before pregnancy and 18.2% during pregnancy. Smoking during pregnancy was significantly more prevalent among Spanish women (21.9%) than immigrants (8.7%). Immigrant women were more exposed to second-hand smoke (SHS) at home and at work, lived with more smokers, had longer daily SHS exposure (3.5 vs. 2.6 h), and were more often employed in hospitality. Factors associated with continued smoking included being Spanish, higher daily cigarette consumption, greater SHS exposure, low risk perception, and a partner with lower education	Country or community of origin; Socioeconomic status (poverty, education, employment)
			Knowledge of harms of smoking towards foetus while pregnant			
			A:			
			Attitudes of the risk smoking poses to the foetus			
			B:			
			Tobacco use status and cigarette smoking frequency, as well as nicotine dependence			
Harris 2012 [73]	Bosnian Refugees	Self-identification as Bosnian	K:	Cigarettes	66.4% were current smokers. Current smokers had lower agreement with health risks of smoking and perceived their own risk of heart disease and lung cancer as lower than other smokers. Over half (57.4%) wanted to quit, but only 30.5% had attempted cessation in the past year, with minimal use of cessation aids	Socioeconomic status (poverty, education, employment); Acculturation
			Health risk perception of smoking			
			A:			
			Attitudes to quit smoking			
			B:			
			Smoking behaviour, intensity and current cigarette smoking status			
Giuliani 2012 [74]	Somali refugees	Not explicitly defined	K:	Cigarettes; Waterpipe/shisha	25.7% were current tobacco users—44.1% of men and 3.7% of women. Ever smoking was linked to male gender, some college education, and having close friends who smoked. Nearly all smokers underestimated health risks from Low-level smoking, and 93% of current smokers wanted to quit—but only 42% had tried, and few were willing to use cessation aids or programs. Strong belief in Islamic prohibition of smoking was protective against use	Country or community of origin; Socioeconomic status (poverty, education, employment); Social dynamics (stigma, cultural practice/rituals); Sex/gender (male vs female); Neighborhood residence (Community access to health care), Years in Host Country, Marital status
			Knowledge towards the consequences of tobacco use			
			A:			
			Attitudes towards the consequences of tobacco use, cultural values and, and exposure to tobacco use			
			B:			
			Tobacco use behaviours, cessation behaviours			
FitzGerald 2015 [75]	Chinese immigrants	Chinese descent (either Mandarin orCantonese speaking) and be immigrants to Canada or children of Chinese immigrants	K:	Cigarettes	Among 167 Chinese immigrant smokers (82% male), 45% started smoking due to social factors. While most recognized health risks, younger smokers (< 35 years) were more likely to smoke around children and dismiss secondhand smoke dangers. lower educated smokers believed they would benefit more from smoking than better educated smokers. Barriers included lack of culturally and linguistically appropriate cessation resources and support from care providers	Socioeconomic status (poverty, education, employment); Social dynamics (stigma, cultural practice/rituals); Subjective individual factors (social identity, behavioural factors); Sex/gender (male vs female), peer pressure,
			Smoking-related knowledge of health consequences			
			A:			
			Smoking beliefs, perceptions and attitudes, such as sociocultural and environmental factors, social identity, psychosocial factors			
			B:			
			Current smoker status			
Ezika 2014 [76]	African immigrants	Participants are described as Africans and that"those that participated in the study appear to come from Sub-Saharan Africa"	K:	Cigarettes	The participants’ smoking habits were influenced by cold weather environment as well as societal norms that appear to make the smoking habit more acceptable in Glasgow than Africa. It appears the more educated the participants were, the fewer cigarettes they smoked. Awareness of health risks was average, but most dismissed smoking cessation programs as irrelevant, believing they could quit unaided	Country or community of origin; Socioeconomic status (poverty, education, employment)
			Health risk awareness, Addiction beliefs			
			A:			
			Cessation attitude, social norms, reasons the participants are motivated to smoke, psychological motivation, adventurous motivation to smoke			
			B:			
			Smoking status, frequency			
Charoenca 2021 [77]	Myanmar migrant workes	Born in Myanmar	K:	Cigarettes; Oral tobacco (e.g. snus)	90% were current smokers, predominantly young males (94.3%). Over 90% smoked Daily, started smoking around age 18.5, and favored manufactured or hand-rolled cigarettes. Smoking was strongly linked to social settings and peer networks, while stress levels were generally low. Smokers had moderate knowledge of tobacco harms but held mixed attitudes, seeing smoking as socially beneficial despite health risks	Socioeconomic status (poverty, education, employment); Social dynamics (stigma, cultural practice/rituals); Subjective individual factors (social identity, behavioural factors); Sex/gender (male vs female), psychosocial factors, Marital status, age of initiation of tobacco use, duration of smoking, settings for smoking
			Correct knowledge about tobacco use based on the rating of factual statements			
			A:			
			Number and percentage of positive/negative attitudes on tobacco use			
			B:			
			Smoking pattern and behaviour of smokers			
Pinsker 2017 [78]	Somali Immigrants	Self-identification as Somali	K:	Cigarettes; Waterpipe/shisha; Oral tobacco (e.g. snus)	Somali youth predominantly viewed tobacco use as culturally and religiously inappropriate, driven by peer influence. Misinformation about hookah was present, as it was not seen as tobacco consumption. Prevention strategies suggested by youth included peer-led education, visual media, and social activities. Three culturally tailored videos were co-created and positively received, with high ratings (median scores: 85–94). Youth found the videos informative and effective, particularly when shared through social media, though some preferred private sharing to avoid embarrassment	Social dynamics (stigma, cultural practice/rituals); Subjective individual factors (social identity, behavioural factors)
			Knowledge of Somali youth tobacco use			
			A:			
			Attitudes towards tobacco prevention messaging			
			B:			
			Somali youth tobacco use			
D’Silva 2016 [79]	Migrants	Described as migrant workers residing in a"migrant area"	K:	Cigarettes; Oral tobacco (e.g. snus)	27.7% used tobacco, exclusively among males. None of the females reported any use. Smokeless tobacco was more common (78%) than smoking. Tobacco use was associated with parental and peer tobacco habits. 30.6% had average, and 25.6% had bad knowledge of tobacco’s harms. Many held permissive attitudes toward tobacco use, as only 11.7% believed that tobacco use should be banned in public places	Socioeconomic status (poverty, education, employment); Social dynamics (stigma, cultural practice/rituals); Subjective individual factors (social identity, behavioural factors); Sex/gender (male vs female), Tobaco family habits
			Knowledge on the addictive, harmful nature of tobacco use			
			A:			
			Attitudes towards smoking giving social status, tobacco releases tension, whether adolescents should be discouraged, banned in public places, and attitudes towards tobacco advertising			
			B:			
			Current tobacco use in any form			
Lu 2015 [80]	Immigrants; Foreign-borns	The population is described as immigrants, with all but one being foreign-born who migrated to the US	K:	Cigarettes	Study 1 (Qualitative) identified seven categories of Chinese American immigrant smokers based on the Transtheoretical Model stages and found that risk perception and emotions (fear, worry, disgust) were key in shaping smoking decisions more than uncertainty or communication Study 2 (Quantitative) used latent class analysis and structural equation modeling to show that risk perception, acculturation, social norms, and emotional responses (e.g., fear, disgust) significantly influenced smoking behaviors and nonsmokers’ responses to secondhand smoke exposure. Communication frequency and efficacy were also predictors	Acculturation
			Reflections on risk perception, chronic consequences of smoking (such as knowledge of severe consequences e.g. lung cancer). Myths about second-hand smoke			
			A:			
			Optimistic bias towards smoking underestimated susceptibility			
			B:			
			Immigration experience and smoking decision change. Reflections on smoking history			

Knowledge of tobacco harms was generally high but varied significantly by socio-economic status. While most participants recognised the dangers of smoking, studies found awareness to be significantly lower among current smokers and those with less access to education or health literacy, lower-acculturated or linguistically isolated groups [61, 70, 74]. Misconceptions about specific products—such as hookah, bidis, and smokeless tobacco—were widespread, often perceived as less harmful or even beneficial in certain cultural contexts (e.g., gutkha among South Asians, or hookah among Somali youth) [63, 68, 78].

Attitudes were shaped by acculturation, gender, and religion. Smoking was often seen as masculine, social, or stress-relieving, particularly among African [64, 65], Chinese [60, 71], and Latino [61] men. In contrast, smoking was stigmatised among women, with gender norms restricting both use and access to cessation support [60, 74]. Religious beliefs (e.g., Islam’s prohibition) acted as protective factors, especially among Somali populations [74, 78]. Reported behaviours covered both smoking status and cessation practices. Across the 25 KAB studies, smoking prevalence among migrant populations varied widely, ranging from as low as 2% among women [70] to 94% among male factory workers [77]. Men consistently reported higher smoking rates than women [59, 68, 72, 74, 79]. Smoking was commonly initiated during adolescence or early adulthood, sustained by peer influence, stress, and occupational or migration-related pressures [59, 76, 79]. Quit attempts were common [70, 74], but frequently undermined by limited awareness of cessation services, reliance on willpower [76], or lack of culturally appropriate resources [60, 72]. Notably, acculturated migrants were more likely to seek help [61, 72]. Across conflict-affected populations [59] and factory workers [57, 62], stress and social context were dominant behavioural drivers. Finally, multiple studies emphasised family and peer environments—both risk-enhancing [60, 79] and protective [64]—as critical leverage points for tailored interventions. Intersections between tobacco-related KAB and migrant experiences were further shaped by how studies defined and categorised migrant populations.

#### Terminology of migrants and tobacco products

The analysis revealed wide variability in how migrant groups were defined and studied (see also Table 4). For instance, Castellanos [61], Harris et al. [73], and Pinsker et al. [78] assessed immigrant status through self-identification. Two studies from Wilhelm et al. [63] and Wilhelm et al. [64] assessed Somali refugees, as those who reported foreign birth and identified as ethnically Somali, while the Somali refugees in Giuliani et al. [74] evaded an explicit definition. Some studies used even broader and less precise terms, such as “African immigrants” in Ezika [76], who were described as Africans, and that “those that participated in the study appear to come from Sub-Saharan Africa [68].” Another example includes “migrant workers” in the study by D’Silva [79] who were solely described as residing in a “migrant area” [74]. In addition to variations in defining migrant groups, there were inconsistencies in how the consumption of tobacco and/or nicotine products was addressed across studies. Among the 25 KAB studies, 23 focused on one or more tobacco products, specifically cigarettes (18 studies) and/or water pipe/shisha or oral tobacco (8 studies). However, only Onigbogi et al. [66] specified a particular type of cigarette (menthol cigarettes) [66]. Furthermore, two studies did not clearly define the products considered under “smoking” [70, 71], and five studies mentioned tobacco consumption without specifying, or differentiating between tobacco products [57, 63–65, 81].

## Discussion

Our study is the first to review the global landscape of research on tobacco and nicotine knowledge, attitudes, and behaviour among migrants, highlighting variations in study design, geographical focus, and the terminology used to describe migrant populations and use of tobacco and/or nicotine products.

### Study design and KAB focus

The results indicated that most research on the consumption of tobacco and/or nicotine products amongst migrants is predominantly quantitative and prevalence-behaviour based, with a focus on tobacco cigarettes. This trend seems reasonable as prevalence rates of smoking can be easily established and commonly requested in a questionnaire, such as with simple yes/no questions. This information typically requires fewer resources and less time compared to more in-depth qualitative research that explores knowledge and attitudes of specific migrant groups, which is similarly reflected in our findings of the relatively lower number of these studies. While tobacco smoking prevalence data can provide practical and actionable insights for public health initiatives, the current emphasis on these data highlights the need for a more balanced approach, which can offer valuable insights into the social and cultural contexts influencing these behaviours. For instance, migrants may have diverse reasons for migrating depending on the country of origin, or personal aspirations, but more importantly, they might have different responsiveness to public health interventions in their host country [82, 83]. Cultural, religious, or socio-economic differences might also dictate differences in tobacco and/or nicotine product uptake [84]. For instance, Pinsker et al. [78] showed that the Somali culture and religion prohibit “smoking”, yet hookah use is seen as a way to get people together [78]. Moreover, our results showed that migrant men consistently showed higher smoking rates across studies, often shaped by norms that link smoking with masculinity, stress relief, or social status (especially in regard to menthol cigarettes). While migrant women tended to have lower rates, they also experienced restrictions in accessing cessation aid. These patterns are not just statistical artefacts—they point to deeper gender roles and expectations that shape behaviour and access to help [85]. Understanding the combination of knowledge, attitudes, and behaviours related to the consumption of different tobacco and nicotine products within specific migrant communities is key. This insight enables the development of culturally relevant education campaigns, tailored cessation programmes, and strategic resource allocation to address high-risk groups [86].

### Geographical coverage of the study landscape

Our results show that most studies focused on single host countries. Notably, the USA emerged as the top country either directly or indirectly researching the consumption of tobacco and/or nicotine products by migrant populations. This prominence may be partly attributed to the political support in the 1960s for evaluating the consequences for tobacco use, which marked a pivotal moment in epidemiological tobacco research [87]. The US National Cancer Institute would become one of the most well-funded research institutions globally, supporting a broad portfolio of tobacco control research [53]. Additionally, the USA is home to more international migrants than any other country, with a total of 50.6 million, according to the Global Migrant Data Portal [52]. This highlights the USA’s significance as a global destination for migrants and reflects the high number of studies originating from the USA, which has contributed to the identification of important phenomena, such as the 'hispanic paradox', also known as the immigrant health advantage, referring to the findings that despite facing socioeconomic disadvantages, Hispanic immigrant populations in the U.S. exhibit better health outcomes than non-Hispanic whites [88, 89].

Other countries such as Germany, Canada, China, Australia and India also ranked among the top ten in producing research on the consumption of tobacco and/or nicotine products by migrants, which aligns with the presence of over 35 million international migrants combined in these nations [90]. Conversely, Saudi Arabia, despite having the third highest number of international migrants, had few studies in the literature on this topic. This could be a result of several factors such as, that effective national health research on migrant populations requires collaboration and sufficient funding, often competing with other public health priorities. This may, to some extent, explain the relatively lower output from certain regions. However, it is not just a matter of collaboration and funding; these disparities in research output reflect broader challenges faced by some regions in prioritising and supporting health research on migrant populations. As a result, the global trends in tobacco and nicotine consumption studies we found underline the uneven global differences in research productivity across countries.

The WHO Global Research Agenda on Health, Migration, and Displacement has identified research amongst migrants as a strategic priority [91]. The WHO has emphasised that migration-related research is essential, recognising the significant differences between high-and low-income countries, as well as regional variations. These discrepancies highlight the need for increased research efforts in areas with relatively low output.

### Terminology

We found that the most frequently used terms were “migrants” and “immigrants”. However, current research often lacks clarity on the distinction between these two terms and their relationship to other, less frequently used terms, such as “foreign-born”. A clear distinction between terms, along with explicit definitions and explanations for the choice of terminology in studies, is often absent.

This lack of international consensus stems from the complexities of semantics across different contexts. Technical definitions, concepts, and categories of migrants are shaped by various factors, including geographic, legal, political, methodological, and temporal considerations. The absence of a consensus on other migrant typologies, such as short-term migrant workers, asylum seekers, or undocumented migrants, has also been identified as creating challenges in standardised assessment of international migration. For instance, Douglas et al. [92] explain that migrants as a group can be defined from multiple perspectives, including legal, administrative, and statistical viewpoints. They can be classified based on reasons for migration, such as economic opportunities or safety, and by factors like birthplace, citizenship, or length of stay [92]. Douglas et al. [92] aptly titled their work, “Definitions Matter”, since clear and consistent definitions can guide effective public health interventions, and in context of this scoping review, help develop tobacco and/or nicotine prevention efforts [92]. Delivering effective tobacco and nicotine public health services for migrants is challenging because migrant communities are diverse, and a “one-size-fits-all” approach is not effective [86]. By carefully differentiating between migrant groups and understanding their unique reasons for migration, public health services can be better tailored to meet their specific needs and expectations after resettlement [25, 93]. For instance, research has shown that migrants from Eastern European countries often have high smoking rates and significant exposure to second-hand smoke, which could be influenced by cultural practices or the tobacco control policies in their home countries [25, 94]. Additionally, recognising the impact of forced migration—due to conflict, persecution, or poor working conditions—on stress and smoking may further inform targeted prevention and intervention strategies [95].

### Tobacco and/or nicotine products

Of the 25 KAB studies analysed, 23 focused on specific tobacco products like cigarettes and water pipe/shisha or oral tobacco, while 7 studies lacked clarity on the products included under smoking or tobacco consumption. While the focus on cigarettes and related tobacco products reflects a recent public health focus, the lack of attention to more recent products is concerning. Today, diverse tobacco and nicotine products are available worldwide, including heated tobacco products, disposable e-cigarettes, and nicotine pouches. This diversity and wide array of choices similarly impact the KAB towards smoking, as migrants may move to countries where more, or fewer products are available on the market. Moreover, country-specific, industry marketing descriptions and word choice for more recent tobacco and nicotine products can have implications for how these products are perceived, and subsequently used [96]. The lack of research on more recent products thus will pose challenges for public health, complicating comparisons between settings, countries, and groups. Future KAB research should include more recent products to address these gaps and challenges.

### Further research

This scoping review identifies several important areas for future research on tobacco and/or nicotine use among migrants. First, studies should better integrate KAB to understand how these elements interact and shape habits across diverse migrant groups. Additionally, more qualitative research is needed to explore the cultural, social, and psychological contexts of tobacco and/or nicotine use, as these factors are often overlooked. Expanding research to underrepresented regions, such as the Middle East, South Asia, and Africa, where large migrant populations reside, is also essential. Moreover, with the rise of emerging tobacco and nicotine products like heated tobacco products, e-cigarettes, and nicotine pouches, understanding migrants' KAB is increasingly important. Standardising terminology and definitions across studies would improve data comparability and enhance cross-country research. Longitudinal studies tracking changes in behaviour over time, particularly in relation to migration and acculturation, are also central. Finally, evaluating the effectiveness of culturally tailored public health interventions is necessary to ensure that prevention programmes meet the unique needs of different migrant populations.

### Limitations and strengths

Our methodology only captured published literature in academic journals and excluded the grey literature which could also be a source of information that addresses our review question. This was done however to ensure the quality of our data. Another limitation was the variability in terminology used across different studies, particularly a lack of standardised terminology for describing migrant populations. In this scoping review, we identified 9 different terms used, such as immigrants, refugees, asylum seekers, or displaced persons. Different authors used varying terms to describe similar groups, or vice versa, using the same term to describe different groups, leading to inconsistencies in the literature. This lack of uniformity in terminology could result in the exclusion of relevant studies that used terms not captured by our search strategy. Similarly, when definitions were absent, we still included the study for review if it explicitly mentioned one of the terms, we deemed relevant under the umbrella definition of IOM. Therefore, while the IOM definition guided our work, it also required us to interpret and make decisions about which studies and terms were applicable. Nevertheless, by adhering to the IOM's definition and carefully considering a spectrum range of terms related to “migrants”, we were able to include as many relevant studies as possible for the review and subsequent analysis. Furthermore, the involvement of at least two reviewers during the screening and extraction phases of this review served as a quality assurance approach to ensure there is shared understanding in the information being collected despite the variations in the terminology.

## Conclusions

This scoping review has mapped the global literature on migrant smoking, identifying research gaps and patterns in KAB toward tobacco and nicotine use among various migrant populations. We found a diverse body of work, but several critical gaps emerged, including an underrepresentation of qualitative studies, inconsistent terminology, and a focus skewed toward high-income countries. Our findings highlight the need for more research in low- and middle-income regions, as well as a standardised approach to migrant-related terminology. Additionally, the emphasis on behaviour-focused studies points to opportunities for further exploration of knowledge and attitudes shaping tobacco and/or nicotine use. Addressing these gaps is essential for developing culturally relevant public health strategies. Ultimately, this review highlights the importance of collaborative, cross-border research to inform effective interventions and policies tailored to the complexities of global migration and health.

## Supplementary Information

Supplementary Material 1.

Supplementary Material 2.

Supplementary Material 3.

Supplementary Material 4.

Supplementary Material 5.

Supplementary Material 6.

## Data Availability

Data is provided within the manuscript and supplementary files. All of the papers reviewed in this study are included in Additional file 5.
