# Supplementary material for: Knowledge, attitudes, and behaviours towards smoking among people with migration experience: a global scoping review
Source: BMC Public Health. 2025 Sep 30;25:3217. doi: 10.1186/s12889-025-24258-y (PMC12486493; doi:10.1186/s12889-025-24258-y)
Supplement: Supplementary file 2 — Supplementary Material 2. [file 12889_2025_24258_MOESM2_ESM.pdf]

**Full details of the search strategies used in this Scoping Review**

**Medline ALL Ovid**

(("Transients and Migrants"/ or Refugees/ or exp Human Migration/ or exp "Emigrants and Immigrants"/ or Minority Groups/ or Minority Health/ or "Ethnic and Racial Minorities"/ or Cross-Cultural Comparison/ or Cultural Diversity/ or Vulnerable Populations/ or (migrant* or immigrant* or (migration* adj3 (human* or background*)) or immigration* or emigrant* or "labour migration*" or "labor migration*" or refugee* or asylum* or (forced adj (migra* or displace* or immigra*)) or ((displaced or undocumented) adj3 (person* or people* or population* or women or men or adolescen* or individual*)) or (minorit* adj3 (group* or health* or population*)) or ((underrepresented or under-represented or racial) adj3 (group* or minorit*)) or ((racial* or race) adj3 divers*) or foreigner* or foreign-born* or foreignborn* or multi-ethnic* or multiethnic* or (ethnic* adj3 (difference* or divers* or background* or group* or population* or identit* or minorit*)) or (cultur* adj3 (difference* or divers* or background* or identit* or minorit*)) or CALD or diaspora* or alien* or resettlement* or re-settlement* or (border* adj1 crossing*) or newcomer* or naturalized citizen* or non-native* or nonnative* or "international student*").ti,ab,kf.) and (((exp Smoking/ or "Tobacco Use"/ or "Tobacco Use Disorder"/ or smoking devices/ or electronic nicotine delivery systems/ or smoking pipes/ or smoking water pipes/ or tobacco products/ or tobacco, smokeless/ or tobacco, waterpipe/) and (epidemiology or ethnology or psychology or pc).fs.) or (smoking or smoke or smoker* or tobacco* or cigar or cigars or cigarillo* or cigarette* or pipe or pipes or waterpipe or waterpipes or e-cig* or ecig* or "electr* cigar*" or nicotine or vaporizer or vapourizer or vaporiser or vapouriser or vaper or vapers or vaping or vape or cannabis or snuff or shisha* or snus or "nicotine pouches" or Dokha* or Hooka*).ti,kf. or ((behav* or knowledge* or attitude*) and smoking).ab.) and (Attitude/ or Attitude to Health/ or Awareness/ or Health knowledge, attitudes, practice/ or Health Behavior/ or Health Education/ or Health Literacy/ or Acculturation/ or (attitud* or adapt* or assimilat* or accultur* or aware* or behav* or belief or beliefs or choice* or comprehension or consum* or habits or habit or habitual or knowledge* or meaning or perspective* or practice* or purchas* or pattern* or perception* or perceiv* or prefer* or reflecti* or select* or understanding*).ti,ab,kf.)) not (exp animals/ not humans/) not (letter or news or comment or editorial or congress).pt. not (clinical trial or controlled clinical trial or randomized controlled trial).pt. not trial.ti.

limit 1 to yr="2012 -Current"

**Embase.com**

('migrant'/exp or 'migration'/exp or 'emigrants'/de or 'immigrants'/de or 'minority group'/de or 'minority health'/de or 'ethnic group'/de or 'ancestry group'/de or 'ethnic difference'/de or 'cultural diversity'/de or 'vulnerable population'/exp or (migrant* or immigrant* or (migration* NEAR/3 (human* or background*)) or immigration* or emigrant* or 'labour migration*' or 'labor migration*' or refugee* or asylum* or (forced NEXT/1 (migra* or displace* or immigra*)) or ((displaced or undocumented) NEAR/3 (person* or people* or population* or women or men or adolescen* or individual*)) or (minorit* NEAR/3 (group* or health* or population*)) or ((underrepresented or under-represented or racial) NEAR/3 (group* or minorit*)) or ((racial* or race) NEAR/3 divers*) or foreigner* or 'foreign-born*' or foreignborn* or 'multi-ethnic*' or multiethnic* or (ethnic* NEAR/3 (difference* or divers* or background* or group* or population* or identit* or minorit*)) or (cultur* NEAR/3 (difference* or divers* or background* or identit* or minorit*)) or CALD or diaspora* or alien*

or resettlement* or 're-settlement*' or (border* NEAR/1 crossing*) or newcomer* or 'naturalized citizen*' or 'non-native*' or nonnative* or 'international student*'):ti,ab,kw) **AND** ('smoking and smoking related phenomena'/exp/mj or 'tobacco use'/exp/mj or 'tobacco dependence'/mj or 'smoking device'/exp/mj or 'tobacco'/mj or 'smokeless tobacco'/exp/mj or 'waterpipe tobacco'/mj OR 'smoking habit'/mj or (smoking or smoke or smoker* or tobacco* or cigar or cigars or cigarillo* or cigarette* or pipe or pipes or waterpipe or waterpipes or 'e-cig*' or ecig* or 'electr* cigar*' or nicotine or vaporizer or vapourizer or vaporiser or vapouriser or vaper or vapers or vaping or vape or cannabis or snuff or shisha* or snus or 'nicotine pouches' or Dokha* or Hooka*):ti,kw or ((behav* or knowledge* or attitude*) and smoking):ab) **AND** ('attitude to health'/de or 'awareness'/de or 'health behavior'/de or 'health education'/de or 'health literacy'/exp or 'cultural factor'/de or 'smoking habit'/de OR (attitud* or adapt* or assimilat* or accultur* or aware* or behav* or belief or beliefs or choice* or comprehension or consum* or habits or habit or habitual or knowledge* or meaning or perspective* or practice* or purchas* or pattern* or perception* or perceiv* or prefer* or reflecti* or select* or understanding*):ti,ab,kw) **AND** [2012-2024]/py **NOT** ([animals]/lim NOT [humans]/lim) NOT ([Conference Abstract]/lim OR [Letter]/lim OR [Note]/lim OR [Editorial]/lim) NOT ('clinical trial'/de OR 'controlled clinical trial'/de OR 'randomized controlled trial'/de) NOT (trial):ti

**Web of Science Core Collection** (via Clarivate)

Science Citation Index Expanded (1900-present); Social Sciences Citation Index (1900-present); Arts & Humanities Citation Index (1975-present); Conference Proceedings Citation Index-Science (1990-present); Conference Proceedings Citation Index-Social Science & Humanities (1990-present); Emerging Sources Citation Index (2018-present)

TS=((migrant* or immigrant* or (migration* NEAR/3 (human* or background*)) or immigration* or emigrant* or "labour migration*" or "labor migration*" or refugee* or asylum* or (forced NEAR/1 (migra* or displace* or immigra*)) or ((displaced  or undocumented) NEAR/3 (person* or people* or population* or women or men or adolescen* or individual*)) or (minorit* NEAR/3 (group* or health* or population*)) or ((underrepresented or under-represented or racial) NEAR/3 (group* or  minorit*)) or ((racial* or race) NEAR/3 divers*) or foreigner* or "foreign born*" or foreignborn* or multiethnic* or multi-ethnic* or (ethnic* NEAR/3 (difference* or divers* or background* or group* or population* or identit* or minorit*)) or (cultur* NEAR/3 (difference* or divers* or background* or identit* or minorit*)) or CALD or  diaspora* or alien* or resettlement* or "re-settlement*" or (border* NEAR/1 crossing*) or newcomer* or "naturalized citizen*" or "non native*" or nonnative* or "international student*")) **AND** TS=((smoking or smoke or smoker* or tobacco* or cigar or cigars or cigarillo* or cigarette* or pipe or pipes or waterpipe or waterpipes or e-cig* or ecig* or (electr* NEAR/1 cigar*) or nicotine or vaporizer or vapourizer or vaporiser or vapouriser or vaper or vapers or vaping or vape or cannabis or snuff or shisha* or snus or "nicotine pouche*" or Dokha* or Hooka*)) **AND** TS=((attitud* or adapt* or assimilat* or accultur* or aware* or behav* or belief or beliefs or choice* or comprehension or consum* or habits or habit or habitual or knowledge* or meaning or perspective* or practice* or purchas* or pattern* or perception* or perceiv* or prefer* or reflecti* or select* or understanding*)) AND PY=(2012-2024) NOT TI=(trial)

**APA PsycINFO** (Ovid)

((exp Human Migration/ or Refugees/ or Asylum Seeking/ or Immigration/ or exp Migrant Workers/ or Minority Groups/ or "Racial and Ethnic Differences"/ or "Racial and Ethnic Groups"/ or Cross Cultural Differences/ or Cultural Diversity/ or (migrant* or immigrant* or (migration* adj3 (human* or background*)) or immigration* or emigrant* or "labour migration*" or "labor migration*" or refugee* or asylum* or (forced adj (migra* or displace* or immigra*)) or ((displaced or undocumented) adj3 (person* or people* or population* or women or men or adolescen* or individual*)) or (minorit* adj3 (group* or health* or population*)) or ((underrepresented or under-represented or racial) adj3 (group* or minorit*)) or ((racial* or race) adj3 divers*) or foreigner* or foreign-born* or foreignborn* or multi-ethnic* or multiethnic* or (ethnic* adj3 (difference* or divers* or background* or group* or population* or identit* or minorit*)) or (cultur* adj3 (difference* or divers* or background* or identit* or minorit*)) or CALD or diaspora* or alien* or resettlement* or re-settlement* or (border* adj1 crossing*) or newcomer* or naturalized citizen* or non-native* or nonnative* or "international student*").ti,ab,id.) **AND** (exp Tobacco Smoking/ or "Tobacco Use Disorder"/ or (smoking or smoke or smoker* or tobacco* or cigar or cigars or cigarillo* or cigarette* or pipe or pipes or waterpipe or waterpipes or e-cig* or ecig* or "electr* cigar*" or nicotine or vaporizer or vapourizer or vaporiser or vapouriser or vaper or vapers or vaping or vape or cannabis or snuff or shisha* or snus or "nicotine pouches" or Dokha* or Hooka*).ti,id. or ((behav* or knowledge* or attitude*) and smoking).ab.) **AND** (Attitudes/ or Health Attitudes/ or Awareness/ or Health Awareness/ or Health Behavior/ or Health Education/ or Health Literacy/ or Acculturation/ or (attitud* or adapt* or assimilat* or accultur* or aware* or behav* or belief or beliefs or choice* or comprehension or consum* or habits or habit or habitual or knowledge* or meaning or perspective* or practice* or purchas* or pattern* or perception* or perceiv* or prefer* or reflecti* or select* or understanding*).ti,ab,id.)) not (exp animals/ not humans/) not (letter or comment or editorial or abstract).dt. not exp Clinical Trials/ not trial.ti.

limit 1 to yr="2012 -Current"

**Global Health Ovid**

(Migrants/ or Refugees/ or Immigrants/ or Ethnic Groups/ or Minorities/ or Migrant Labour/ or (migrant* or immigrant* or (migration* adj3 (human* or background*)) or immigration* or emigrant* or "labour migration*" or "labor migration*" or refugee* or asylum* or (forced adj (migra* or displace* or immigra*)) or ((displaced or undocumented) adj3 (person* or people* or population* or women or men or adolescen* or individual*)) or (minorit* adj3 (group* or health* or population*)) or ((underrepresented or under-represented or racial) adj3 (group* or minorit*)) or ((racial* or race) adj3 divers*) or foreigner* or foreign-born* or foreignborn* or multi-ethnic* or multiethnic* or (ethnic* adj3 (difference* or divers* or background* or group* or population* or identit* or minorit*)) or (cultur* adj3 (difference* or divers* or background* or identit* or minorit*)) or CALD or diaspora* or alien* or resettlement* or re-settlement* or (border* adj1 crossing*) or newcomer* or naturalized citizen* or non-native* or nonnative* or "international student*").ti,ab,id.) **AND** ((Tobacco Smoking/ or exp Tobacco/ or Cannabis Smoking/ or (smoking or smoke or smoker* or tobacco* or cigar or cigars or cigarillo* or cigarette* or pipe or pipes or waterpipe or waterpipes or e-cig* or ecig* or "electr* cigar*" or nicotine or vaporizer or vapourizer or vaporiser or vapouriser or vaper or vapers or vaping or vape or cannabis or snuff or shisha* or snus or "nicotine pouches" or Dokha* or Hooka*).ti,id.) or ((behav* or knowledge* or attitude*) and smoking).ab.) **AND** (Attitudes/ or Attitudes to Health/ or Awareness/ or Health Behavior/ or Health Education/ or exp Literacy/ or Acculturation/ or (attitud* or adapt* or assimilat* or accultur* or aware* or behav* or belief or beliefs or choice* or comprehension or consum* or habits or habit or habitual or knowledge* or meaning or perspective* or practice* or purchas* or pattern* or perception* or perceiv* or prefer* or reflecti* or select* or understanding*).ti,ab,id.) **NOT** (editorial or correspondence or conference).pt. not (exp clinical trials/) not trial.ti.

limit to yr="2012 -Current"
