# Supplementary material for: Knowledge, attitudes, and behaviours towards smoking among people with migration experience: a global scoping review
Source: BMC Public Health. 2025 Sep 30;25:3217. doi: 10.1186/s12889-025-24258-y (PMC12486493; doi:10.1186/s12889-025-24258-y)
Supplement: Supplementary file 3 — Supplementary Material 3. [file 12889_2025_24258_MOESM3_ESM.pdf]

Full-text screening: Scoping Review
Key inclusion criteria:
Study Identification
Record ID
____

The study's focus is on a migrant population.	Yes<br>No
The study is presenting research on the knowledge, attitudes, and/or behaviours towards smoking.	Yes<br>No
Include study based on full-text.	Yes<br>No
If no and exclude article, provide reason.	• Does not address KAB.<br>• Does not address smoking.<br>• Focus is not on migrant population.<br>• The study population is local, or native to the country (indigenous/ethnic minority)<br>• The study is a clinical trial.<br>• Other,____

Migrant specific population:

For the purpose of this scoping review, we refer to the UN International Organisation for Migration:
Defined a migrant as "an umbrella term, not defined under international law, reflecting the common lay understanding of a person who moves away from his or her place of usual residence, whether within a country or across an international border, temporarily or permanently, and for a variety of reasons.

Knowledge, attitudes, behaviour (KAB) of smoking:

For the scoping review, based on the KAB approach definitions by Schrader & Lawless (2004), we define knowledge (K) as embodying "all information that a person possesses or accrues related to a particular field of study." For smoking, this can be in regard to knowledge of harmful effects, or perception of health effects of smoking (e.g. "smoking is harmful", "smoking can cause heart diseases") (Multani, Reddy, Bhat, & Sharma, 2012).

Attitudes (A) refer to the subjective "sum or aggregate of all feelings and dispositions toward a particular concept, idea or action" (Thurstone, 1928). Attitudes can be a belief or idea associated with a particular object, can represent the individual's evaluation and emotion associated with the object, or attitudes can represent the predisposition of action towards the object (Schrader & Lawless, 2004). Smoking related attitudes include "smoking is

pleasurable", "smoking relaxes me", "smoking helps me lose weight" (Xu, Leung, Li, Wang, & Zhao, 2015).

Behaviour(s) (B) are understood as an observable action. In other words, the way a person, or group of persons act in certain conditions (Schrader & Lawless, 2004). Smoking related behaviours include "tobacco use among Somali adults in Minnesota", "hookah smoking amongst Eritrean adults" (example titles from scoping review library).

Interaction of KAB:
Keep in mind that KAB are likely to overlap. Research indicates that the relationship between K, A, and B is complex, reciprocal, and dynamic. For example, as stated by Schrader & Lawless (2004), "what an individual knows may inform his or her attitude about that topic, and how he or she feels about that topic may influence behaviour (see Figure 1 below of simplified model by Chaffee & Roser (1986) on KAB consistency.)

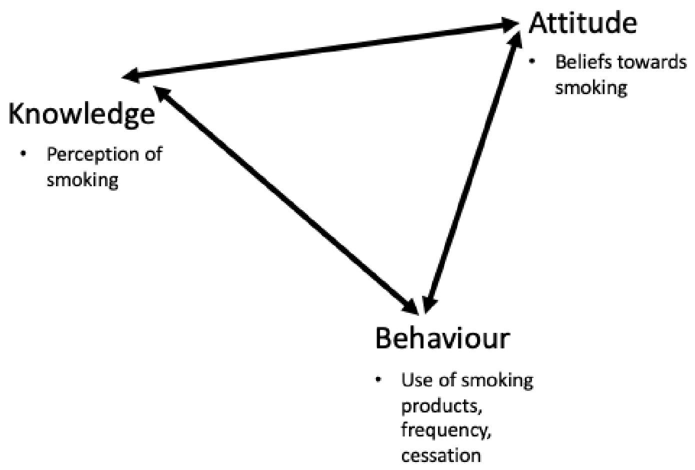

Figure 1 Simplified Model of Knowledge-Attitude-Behaviour Consistency (Chaffee & Roser, 1986)

Smoking: Use of Tobacco and/or nicotine products
For the scoping review, smoking is an umbrella term used to include the consumption of any tobacco and/or nicotine products. Example products include cigarettes, waterpipe/shisha, vapes, e-cigarettes, cannabis (shall be included), snuff, snus, nicotine pouches.

To consider:

- Context of the study – is the study population local, native or indigenous to the country? → If yes, exclude.
- Study population – is this a clinical trial? → If yes, exclude.
    - We are only interested in real world data, so ensure laboratory, clinical trials are excluded.

References:

Chaffee, S. H., & Roser, C. (1986). Involvement and the consistency of knowledge, attitudes, and behaviors. *Communication research*, *13*(3), 373-399.

Multani, S., Reddy, J. J., Bhat, N., & Sharma, A. (2012). Assessment of knowledge, attitude, behaviour and interpersonal factors related to the use of tobacco among youth of Udaipur city, Rajasthan, India: A cross-sectional study. *Addiction & health*, *4*(3-4), 142.

Schrader, P. G., & Lawless, K. A. (2004). The knowledge, attitudes, & behaviors approach how to evaluate performance and learning in complex environments. *Performance Improvement*, *43*(9), 8-15.

Thurstone, L. L. (1928). Attitudes can be measured. *American journal of Sociology*, *33*(4), 529-554.

Xu, X., Leung, D. Y. P., Li, B., Wang, P., & Zhao, Y. (2015). Smoking-related knowledge, attitude, social pressure, and environmental constraints among new undergraduates in Chongqing, China. *International journal of environmental research and public health*, *12*(1), 895-909.
