# Supplementary material for: Knowledge, attitudes, and behaviours towards smoking among people with migration experience: a global scoping review
Source: BMC Public Health. 2025 Sep 30;25:3217. doi: 10.1186/s12889-025-24258-y (PMC12486493; doi:10.1186/s12889-025-24258-y)
Supplement: Supplementary file 4 — Supplementary Material 4. [file 12889_2025_24258_MOESM4_ESM.pdf]

Data Extraction Form Scoping Review

This data extraction form will be used to extract relevant data from the included studies.

Study Details and Characteristics:
Record ID
____

Title of article	
Author(s)	
Year of publication	

Extraction Criteria:

Purpose/Main aim of study	Copy and paste from article
Study design<br>• Data source/Tools (e.g., survey)	Quantitative<br>• Randomized controlled trials (RCTs)<br>• Cluster RCTs<br>• Quasi-experimental studies<br>• Cohort studies<br>• Controlled-before-and-after studies (pre/post-tests).<br>• Cross-sectional<br>• Primary quantitative data<br>• Secondary quantitative data: National survey data Cohort data<br>• Systematic review Other, namely______<br><br>Qualitative<br>• Interview studies<br>• Focus groups<br>• Observations<br>• Other, namely ____________<br><br>Mixed methods
What is the country/countries the study was completed in?	
What is the geographical level of inquiry?	• Continent

	• Country<br>• Region<br>• City<br>• Neighbourhood<br>• Community<br>• Other, namely ______
Population studied characteristics (sample size, age range)	Copy and paste from article
Which migrant specific population(s) are being researched?	• Migrant<br>• Immigrant<br>• Refugee<br>• Asylum seeker<br>• Internally displaced person<br>• Foreign-born<br>• Expat<br>• People of a specific country of origin, namely ______<br>• Other, namely___________
If the study is describing a specific type of population as multiple groups, such as Asian Americans as both foreign-born and immigrants, state here	Copy and paste from article
Is the study focusing exclusively on migrant populations, or also other populations, such as natives?	Exclusively migrant populations<br><br>Migrant and other populations
Rationale/argument for focus on migration	Copy and paste from article if any given
Recruitment method	Copy and paste from article
Is the study an intervention study?	• No<br>• Yes
	• If yes: copy and paste intervention description from article
Is the study considering factors apart from migration, or migration background?<br><br>Select relevant factors.	• Country-or community of origin<br>• Socioeconomic status (poverty, education, employment)<br>• Social dynamics (stigma, cultural practice/rituals)<br>• Subjective individual factors (social identity, behavioural factors)<br>• Lifestyle factors (alcohol)<br>• Sex/gender (male vs female)

	• Community access to health care (neighborhood residence)<br>• Sexual orientation<br>• Intersectionality<br>• Acculturation<br>• Other
Does the study explore knowledge and/or attitudes, and/or behaviours (KAB) towards smoking?<br><br>*Select relevant factors.*<br><br>*See definition of KAB below.*	• Only knowledge<br>• Only attitudes<br>• Only behaviours<br>• Knowledge and attitudes<br>• Knowledge and behaviours<br>• Attitudes and behaviours
How relevant is smoking for the study?<br><br>Select the focus on smoking the study had.	Exclusively on smoking, and/or drawing correlations between smoking and other factors, such as alcohol consumption, or mental health<br><br>On a topic not related to smoking, where smoking is only recorded as part of participant health profile, for example.
Outcomes reported (Indicators, measures…)	Knowledge: Copy and paste from article (For example, multiple choice questionnaire concerning perceptions of smoking harm to health)
	Attitudes: Copy and paste from article (positive attitudes towards smoking, negative attitudes; Factors that influence positive or negative attitudes)
	Behaviours/practices: Copy and paste from article. For prevalences, specifically state that it is just a prevalence (state what type of prevalence, such as yes/no smoker)
	Integrated outcomes, if any: Copy and paste from article
	Contextual/environmental factors that influence knowledge, attitude and/or practices: Copy and paste from article.

Which “smoking” product is the focus of the study?<br><br>Select relevant factors.	• Cigarettes<br>• Menthol Cigarettes<br>• E-Cigarettes (Vapes)<br>• Heated tobacco products (e.g. IQOS)<br>• Waterpipe/shisha<br>• Cannabis<br>• Oral tobacco (snus)<br>• Nicotine pouches (no tobacco)<br>• Pipe tobacco<br>• Other, namely ________

Knowledge, attitudes, behaviour (KAB) of smoking:

For the scoping review, based on the KAB approach definitions by Schrader & Lawless (2004), we define knowledge (K) as embodying "all information that a person possesses or accrues related to a particular field of study." For smoking, this can be in regard to knowledge of harmful effects, or perception of health effects of smoking (e.g. "smoking is harmful", "smoking can cause heart diseases") (Multani, Reddy, Bhat, & Sharma, 2012).

Attitudes (A) refer to the subjective "sum or aggregate of all feelings and dispositions toward a particular concept, idea or action" (Thurstone, 1928). Attitudes can be a belief or idea associated with a particular object, can represent the individual's evaluation and emotion associated with the object, or attitudes can represent the predisposition of action towards the object (Schrader & Lawless, 2004). Smoking related attitudes include "smoking is pleasurable", "smoking relaxes me", "smoking helps me lose weight" (Xu, Leung, Li, Wang, & Zhao, 2015).

Behaviour(s) (B) are understood as an observable action. In other words, the way a person, or group of persons act in certain conditions (Schrader & Lawless, 2004). Smoking related behaviours include "tobacco use among Somali adults in Minnesota", "hookah smoking amongst Eritrean adults" (example titles from scoping review library).

Interaction of KAB:
Keep in mind that KAB are likely to overlap. Research indicates that the relationship between K, A, and B is complex, reciprocal, and dynamic. For example, as stated by Schrader & Lawless (2004), "what an individual knows may inform his or her attitude about that topic, and how he or she feels about that topic may influence behaviour (see Figure 1 below of simplified model by Chaffee & Roser (1986) on KAB consistency.)

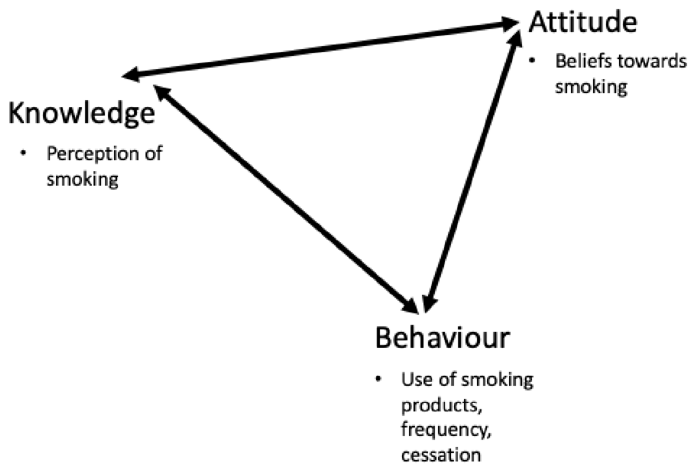

Figure 1 Simplified Model of Knowledge-Attitude-Behaviour Consistency (Chaffee & Roser, 1986)

References:

Arshad, A., Matharoo, J., Arshad, E., Sadhra, S. S., Norton-Wangford, R., & Jawad, M. (2019). Knowledge, attitudes, and perceptions towards waterpipe tobacco smoking amongst college or university students: a systematic review. *BMC Public Health*, *19*, 1-11.

Aslany, M., Carling, J., Mjelva, M. B., & Sommerfelt, T. (2021). Systematic review of determinants of migration aspirations. *Changes*, *1*, 18.

Castañeda, H. (2022). *Migration and Health: Critical Perspectives*. Taylor & Francis.

Cattacin, S. (2012). Spielräume und Regulierungen in einer berauschenden Gesellschaft. *EKDF, Eidgenössische Kommission für Drogenfragen (éd.). Drogenpolitik als Gesellschaftspolitik. Ein Rückblick auf dreissig Jahre Schweizer Drogenpolitik. Zürich*, 42-49.

Galea, S., Ettman, C. & Zaman, M. (2022). *Migration and Health*. Chicago: University of Chicago Press. https://doi.org/10.7208/chicago/9780226822495

Carling, J. (2023). The phrase 'refugees and migrants' undermines analysis, policy and protection. *Int Migr*, *61*(3), 399-403.

Ranci, C. (2010). Social vulnerability in Europe. In *Social vulnerability in Europe: The new configuration of social risks* (pp. 3-24). London: Palgrave Macmillan UK.

Selamoglu, M., Erbas, B., Kasiviswanathan, K., & Barton, C. (2022). General practitioners' knowledge, attitudes, beliefs and practices surrounding the prescription of e-cigarettes for smoking cessation: a mixed-methods systematic review. BMC Public Health, 22(1), 1-12.

Von Heyden, M., Jungaberle, H., & Majić, T. (Eds.). (2018). *Handbuch Psychoaktive Substanzen*. Springer Berlin Heidelberg.

Walker, A. K., & Fox, E. L. (2018). Why marginalization, not vulnerability, can best identify people in need of special medical and nutrition care. *AMA journal of ethics*, *20*(10), E941.
